# Square-Planar Heteroleptic Complexes of α-Diimine-Ni^II^-Catecholate Type: Intramolecular Ligand-to-Ligand Charge Transfer

**DOI:** 10.3390/molecules26154622

**Published:** 2021-07-30

**Authors:** Kira I. Pashanova, Vladlena O. Bitkina, Ilya A. Yakushev, Maxim V. Arsenyev, Alexandr V. Piskunov

**Affiliations:** 1 Laboratory of Metal Complexes with Redox-Active Ligands, G.A. Razuvaev Institute of Organometallic Chemistry, Russian Academy of Sciences, 49 Tropinina Street, 603137 Nizhny Novgorod, Russia; pashanova@iomc.ras.ru (K.I.P.); vlada-bit@yandex.ru (V.O.B.); mars@iomc.ras.ru (M.V.A.); 2 N.S. Kurnakov Institute of General and Inorganic Chemistry, Russian Academy of Sciences, 31 Leninski Prospect, 119991 Moscow, Russia; ilya.yakushev@igic.ras.ru

**Keywords:** *o*-benzoquinone, α-diimine, Ni^II^ ion, photoinduced intramolecular charge transfer, π–π stacking, SC X-ray, cyclic voltammetry, UV-Vis-NIR spectroscopy

## Abstract

Two heteroleptic Ni^II^ complexes combined the redox-active catecholate and 2,2′- bipyridine ligand platforms were synthesized to observe a photoinduced intramolecular ligand-to-ligand charge transfer (LL’CT, HOMO_catecholate_ → LUMO_α-diimine_). A molecular design of compound [Ni^II^(3,6-Cat)(bipy)]∙CH_3_CN (**1**) on the base of bulky 3,6-di-*tert*-butyl-*o*-benzoquinone (**3,6-DTBQ**) was an annelation of the ligand with an electron donor glycol fragment, producing derivative [Ni^II^(3,6-Cat^gly^)(bipy)]∙CH_2_Cl_2_ (**2**), in order to influence the energy of LL’CT transition. A substantial longwave shift of the absorption peak was observed in the UV-Vis-NIR spectra of **2** compared with those in **1**. In addition, the studied Ni^II^ derivatives demonstrated a pronounced negative solvatochromism, which was established using a broad set of solvents. The molecular geometry of both compounds can be ascribed as an insignificantly distorted square-planar type, and the π–π intermolecular stacking of the neighboring α-diimines is realized in a crystal packing. There is a lamellar crystal structure for complex **1**, whereas the perpendicular T-motifs with the inter-stacks attractive π–π interactions form the packing of complex **2**. The redox-active nature of ligand systems was clearly shown through the electrochemical study: a quasi-reversible one-electron reduction of 2,2′-bipyridine and two reversible successive one-electron oxidative conversations (“catecholate dianion—*o*-benzosemiquinonato radical anion—neutral *o*-benzoquinone”) were detected.

## 1. Introduction

The numerous class of the charge transfer complexes involves the metal derivatives with a pronounced back donation effect, driving the strong metal-to-ligand transition (MLCT)—fine representatives are the NIR dyes such as Mo [1,2] and Ru compounds [1,2,3]. Considerable attention has also been paid to transition metal chromophores with a predominant metal-to-ligand magnetic exchange. As an example, the strong “metal-ligand” coupling is inherent for tris(*o*-iminosemiquinonato) Fe^III^ complexes [4,5], as well as for bis(*o*-iminosemiquinonato) Cu^II^ compounds which are distorted tetrahedrally and contain the “soft” donor S/Se atoms in additional functions [6,7,8]. High absorptivity of the similar visible/NIR MLCT dyes is caused by an effective overlapping between metal d-orbitals and ligand π-orbitals due to a realization of high coordination numbers or a significant deviation from planarity.

On the contrary, a planar mutual arrangement of the interacting molecular orbitals and, consequently, a planar molecular structure of the compound should be optimal for the implementation of intramolecular ligand-to-ligand charge-transfer (LL’CT) [9,10]. In this way, the main attention is addressed to a molecular design towards derivatives of 10 group metals (Ni, Pd, Pt), preferring a square-planar coordination environment.

**Scheme 1:**
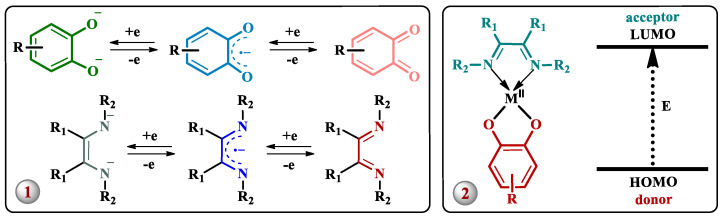
Redox states of *o*-benzoquinone and α-diimine type ligands (**1**); ligand-to-ligand charge transfer HOMO_catecholate_ → LUMO_α-diimine_ (**2**).

The synergistic existence of donor and acceptor organic parts in the coordination sphere of the metal center is a distinctive feature for a wide range of LL’CT chromophores. The acceptor moiety is usually represented by a neutral redox form of α-diimines (Scheme 1) with significant steric hindrances [10,11,12,13,14,15,16,17,18,19,20,21,22,23,24] and/or a rigid carbon skeleton [13,15,25,26,27]. Thus, it provides the required planarity of the complex’s structure. In turn, *o*-quinone [28,29] and related *o*-iminoquinone [29,30,31,32] type derivatives are known donor ligand systems in the charge-transfer complexes. The redox-active nature [29,33,34] of these organics makes it possible to realize their most electronically saturated two-electron reduced redox state (catecholate and amidophenolate, respectively) when such compounds are in the coordination sphere of the metal complex (Scheme 1, (**1**)). The extended possibilities for the functionalization of *o*-quinone/*o*-iminoquinone type ligand systems are a crucial factor that predetermines their widespread use in the generation of the charge-transfer complexes.

**Scheme 2:**
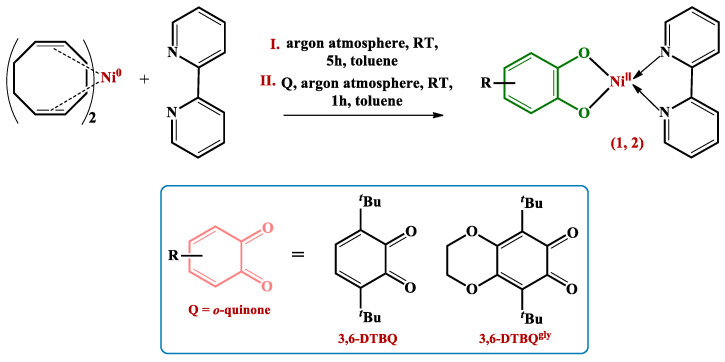
Synthetic route for preparing of **1** and **2**.

Since a charge separation in LL’CT chromophores occurs at the molecular level, it becomes possible to observe the photoinduced intramolecular LL’CT from the HOMO of the electron-saturated donor ligand to the LUMO of the electron-deficient acceptor ligand (Scheme 1, (**2**)). However, in real systems, the HOMO_donor_ → LUMO_acceptor_ assignment of the charge transfer processes can be complicated by the mixed metal/ligand-to-ligand charge transfer [10,19,22,35]. In such cases, the HOMO is formed by the contribution from the metal ion and the donor ligand, while the LUMO is localized on the acceptor organic part.

Changes in the donor/acceptor ability of ligand platforms can affect the charge transfer energy. In particular, an introduction of the acceptor functional groups into the donor ligand should increase the LL’CT energy. Corresponding absorption peaks will shift to the shortwave region of UV-Vis-NIR spectrum, as a consequence. On the contrary, the presence of the donor functions will have the opposite effect.

Despite such variability of the LL’CT process towards α-diimine-M^II^-catecholate compounds, there are sporadic works devoted to a targeted molecular design of LL’CT dyes with the primary goal to influence the position and/or intensity of the electronic absorption bands [10,15,21,22,23,36,37,38], and solvatochromic properties [17,19,22,23,39]. On the other hand, the related square-planar Pd^II^ derivatives are the research objects due to a cytotoxic activity [40,41,42,43]. A series of works describes a modification of the coordination sphere of Pd^II^ ion by non-substituted diimine and catecholate ligand platforms with extended π-system [25,26,27,44,45,46]. It has been recently presented the following noteworthy tendencies in the molecular design of the α-diimine-M^II^-catecholate compounds [13]. Thus, utilization of the conjugated organic ligands provides the π–π stacking, and perfluoroalkylation of a catecholate forces its electron-withdrawing properties to increase the complexes’ stability towards atmospheric oxygen.

Nevertheless, the rich possibilities of molecular design for square-planar LL’CT chromophores were demonstrated in the paper [15]. A substantial change of the LL’CT energy from 1.9 to 0.9 eV (650 nm and 1370 nm in the UV-Vis-NIR spectra, respectively) was achieved successfully. The pronounced solvatochromic effect in a series of the α-diimine–M^II^–catecholate charge transfer complexes (where M^II^ is Ni^II^, Pd^II^, Pt^II^; 3,5-di-*tert*-butyl-catechol, 4,4′-di-*tert*-butyl-2,2′-bipyridine) was described in recent work [17]. The expected negative solvatochromism was established. It has been shown that the nature of the metal center could play a role in LL’CT energy. A slight narrowing of the HOMO–LUMO gap was found within the [Ni] < [Pd] < [Pt] row.

The possibility to control the LL’CT energy in the heteroleptic coordination compounds of the α-diimine–M^II^–catecholate type (M = Ni, Pd, Pt) is an urgent practical issue. These metal complexes and their ionic derivatives exhibit a high absorptivity in the visible and NIR regions due to the realization of LL’CT with 10^3^–10^4^ M^−1^·cm^−1^ molar extinction coefficients. This is one of the main criteria [47] for using of compounds as sensitizers, particularly in photovoltaics. So, different types of coordination compounds with an efficient charge separation have found an application in the dye-sensitized solar cells (DSSCs) [47,48] as “redox mediators” [49] in combination with a solid-state semiconductor surface (usually porous TiO_2_), i.e., in the Grätzel cells [50,51,52]. In particular, pioneer investigations were aimed at testing of the six-coordinated thiocyanato Ru^II^ MLCT chromophores, containing (1) diimines equipped with anchor groups (for instance, phosphonate and carboxylate fragments), (2) ancillary ligands (usually substituted bipyridines and terpyridines) [47,48]. In recent years, a novel trend has spread—using of square-planar LL’CT compounds of Pd [53,54], Pt [22,55,56,57], Cu [49,58], Ni [53,54,59,60] as materials for DSSCs. Research in the field of the molecular design of the square-planar sensitizers is carried out in several directions following existing problems in photovoltaic characteristics. A short lifetime of excited state for the square-planar first-row transition metal complexes can be raised by: (1) using of metal center with a full d-level—such approach was reported for the bis(arylimino)acenaphthene Cu^I^ complexes [58]; (2) creating of a strong crystal field, as in the case of square-planar α-diimine-M^II^-dithiolate species—Ni^II^ [60] and Pt^II^ [57] complexes. Incorporation of electron-withdrawing anchor functions, primarily of a carboxylate type [60], into the diimine fragment facilitates an attachment of sensitizers into the semiconductor surface. Besides, it promotes a redshift of the LL’CT absorption band. It is important for harvesting the sunlight [61] because NIR light is approximately 45% of solar energy [59]). The authors in [15] have recently demonstrated that modifications of the donor *o*-quinone type ligand platforms have a much more noticeable effect on the LL’CT energy than the transformations in the acceptor α-diimine part. For instance, an alternation of the α-diimine moiety with the keeping of the same catecholate part led to a spectral shift Δλ_max_ ≈ 220–350 nm, whereas the varying of catecholate results in a more significant displacement up to Δλ_max_ ≈ 430–550 nm. Thus, a targeted modification of a catecholate part by donor substituents is assumed to be an essential issue to know how a fine-tuning of a catecholate’s composition and electronic structure can change the LL’CT energy.

In the current work, two α-diimine–M^II^–catecholate species **1** and **2** are studied. It should be mentioned that the synthesis of complex **1** was reported in ref. [16] for the first time. Herein, a comprehensive study was performed for compound [Ni^II^(3,6-Cat)(bipy)]∙CH_3_CN (**1**) (where bipy = 2,2′-bipyridine, 3,6-Cat = 3,6-di-*tert*-butyl-catechol) which was prepared in the course of a modified synthetic procedure. The present paper focuses on the molecular, electronic structure and investigates a photoinduced intramolecular LL’CT and solvatochromism. New complex [Ni^II^(3,6-Cat^gly^)(bipy)]∙CH_2_Cl_2_ (**2**) (where 3,6-Cat^gly^—catecholate redox form of 7,10-di-*tert*-butyl-2,5-dioxabicyclo(4.4.0)deca-1(10),6-diene-8,9-dione (**3,6-DTBQ^gly^**) is presented in the same manner. It was shown that an annelation of a quinone type ligand **3,6-DTBQ** by an electron donor glycol fragment could decrease the LL’CT energy substantially.

## 2. Results and Discussion

### 2.1. Synthetic Procedure for the Complexes ***1*** and ***2***

Three known techniques requiring anaerobic conditions or an inert atmosphere (nitrogen, argon) are known for preparing of new bis(ligand) transition metal compounds of the α-diimine–M^II^–catecholate type. The first approach assumes a stepwise synthesis: (1) treatment of the transition metal compound with a quinone type ligand to generate a catecholate-containing intermediate (redox process) [15]; (2) an exchange reaction with the participation of the catecholate precursor and α-diimine. The principal peculiarity of the second method (most frequently used) is in the reverse order of the introduction of ligands into the coordination sphere of the metal center (compared to the first technique) [11,12,13,15,17,19,22,25,35,44,46]. The third combined method is the “one-pot” synthesis [16,36], which was applied to obtain the complex **1** described in ref. [16] for the first time. The synthetic procedure proposed by authors [16] involves Ni(CO)_4_ as a starting metal-containing reactant, requires heating of a reaction mixture (30 min at ~30 °C, and after that 2 h at ~80 °C), and a periodical removing of CO, yielding the targeted product at 42%. In the present work, derivative [Ni^II^(3,6-Cat)(bipy)]∙CH_3_CN was generated by a modified route.

It is a remarkable fact that using of the same diimine and catechol ligands can form not only the square-planar aforementioned Ni^II^ compound (LL’CT chromophore). The six-coordinated complex consists of two 3,6-di-*tert*-butyl-*o*-benzosemiquinone radicals, and a neutral diimine ligand [62] can be obtained too. Such a result was achieved in the course of a reaction between 3,6-di-*tert*-butyl-*o*-benzosemiquinonato Ni^II^ derivative and 2,2′-bipyridine under evacuated conditions. As it can be expected, an effective overlapping of the metal and ligand orbitals was observed in the case of this octahedral complex [62].

Following the second synthetic approach, Ni^II^ complexes **1** and **2** were prepared in two steps (Scheme 2). At the first stage, an interaction between Ni(cod)_2_ and 2,2′-bipyridine was carried out in an inert argon atmosphere with an accompanying turn in a solution’s color from yellow to intense purple. A stoichiometric amount of **3,6-DTBQ** (for **1**) or **3,6-DTBQ^gly^** (for **2**) was added to this reaction mixture at the second stage. A subsequent stirring for about 1 h without heating generated the targeted heteroleptic Ni^II^ derivatives. Single crystals of compounds **1** and **2** were grown from a mixture of CH_2_Cl_2_/CH_3_CN and CH_2_Cl_2_/hexane, respectively, by slow evaporation of the solvents under reduced pressure. The yield of complexes **1** and **2** was 68% and 76%, respectively.

### 2.2. Molecular Structures of Complexes ***1*** and ***2***

Known four-coordinated ligand-to-ligand charge transfer complexes possess a square-planar/slightly distorted square-planar molecular geometry due to a rigid structure of used organic moieties. However, the variability in ligand’s bulkiness and rigidity of the conjugated π-systems determines the several common types of a crystal packing: (1) a lamellar structure [13,35,63,64]; (2) T-shaped perpendicular/herringbone motifs (both cases are accompanied traditionally by π–π* stacking and/or metal-π short contacts [11,14,15,17,25,26,44,45]); (3) a “chaotic” arrangement of molecules without any considerable π–π interactions [16,39] (this type is a relatively rare and observed usually for the complexes bearing aliphatic α-diimines, N-substituted by bulky functions and groups which can rotate freely, such as –C(CH_3_)_3_ [16], –(C_6_H_5_)OMe [39], etc.).

**Figure 1:**
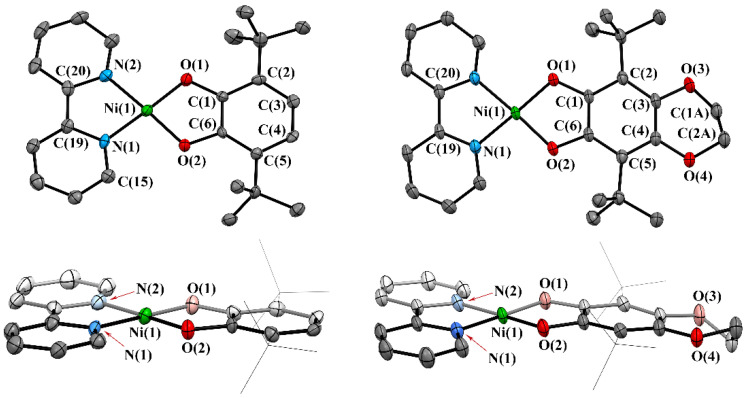
Views of **1** (**left**) and **2** (**right**) molecular structures with 50% thermal probability ellipsoids. Hydrogen atoms are omitted for clarity.

Heteroleptic Ni^II^ complexes **1** and **2** are distinguished by a slightly distorted square-planar geometry of a coordination core (τ_4_(**1**, **2**) = 0.03) [65,66] (Figure 1). The dihedral angle’s values between the O(1)–Ni(1)–O(2) and N(1)–Ni(1)–N(2) planes are minimal and equal to 0.95° (for **1**) and 0.58° (for **2**). In addition, there is a negligible exit of Ni^II^ center from the plane O(1)–O(2)–N(1)–N(2) of a coordination polyhedron—0.007 Å in **1**, and 0.003 Å in **2**. Moreover, the dihedral angles between the planes of 2,2′-bipyridine and a catecholate dianion (O(1)–C(1)–C(2)–C(3)–C(4)–C(5)–C(6)–O(2)) are also small and found at 4.12° and 2.95° (for **1** and **2**, respectively). The C(1A) and C(2A) atoms in a glycol fragment of **2** deviate from a catecholate’s plane at 0.693 Å and 0.120 Å, respectively (Figure 1).

Both studied compounds possess an identical unambiguous electronic structure: a coordination environment of Ni^II^ ion consists of the neutral 2,2′-bipyridine and the catecholate dianion. The bond lengths of α-diimine are in a good agreement with those typical for a neutral ligand, interacting with the metal center through a donor-acceptor manner [13,25,35,46]. A relative equidistance of C–C bonds (1.379(6)–1.422(6) for **1**, 1.400(4)–1.410(5) for **2**), and the values of C–O lengths (1.348(5)–1.365(5) for **1**, 1.360(4)–1.368(4) for **2**) correspond to the distinctive metrical pattern (average C–C = 1.39–1.41 Å, C–O = 1.32–1.39 Å) [29,30] that prove the presence of two-electron reduced catecholate redox form of used *o*-benzoquinones in Ni^II^ derivatives **1** and **2** (Table 1). Confirming a divalent state of the Ni center which is chelated by the catecholate dianion in the square-planar heteroleptic derivatives of similar type, the Ni–O distances are in the intervals at 1.814(3)–1.815(3) Å in **1**, 1.814(3)–1.818(2) Å in **2**. Selected bond lengths and angles are listed in Table 1.

**Figure 2:**
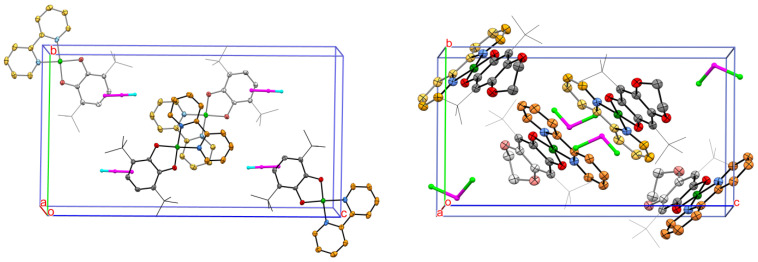
Views of unit cells of **1** (**left**) and **2** (**right**) with 50% thermal probability ellipsoids. Hydrogen atoms are omitted for clarity. Carbon atoms of diimines are orange-colored.

**Figure 3:**
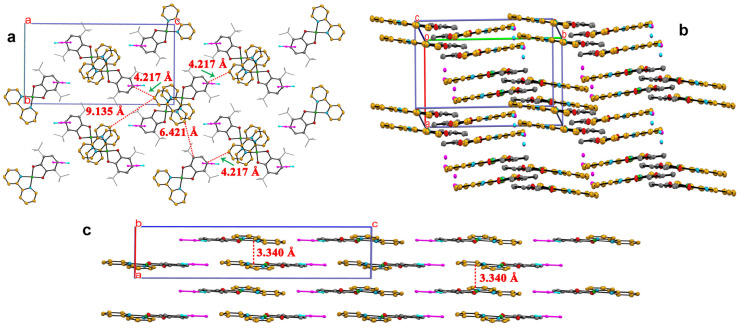
Views of crystal packing fragments of **1** (two unit cells) with 50% thermal probability ellipsoids. *Tert*-butyl groups (**b**) and hydrogen atoms are omitted for clarity. Carbon atoms of diimines are orange-colored. Views (**a**,**c**) demonstrate types of intermolecular contacts.

**Figure 4:**
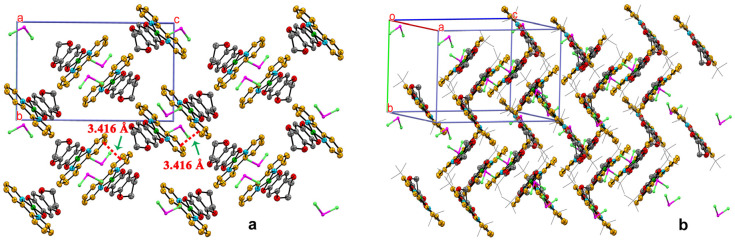
Views of crystal packing fragments of **2** (two unit cells) with 50% thermal probability ellipsoids. *Tert*-butyl groups (view (**a**)) and hydrogen atoms are omitted for clarity. Carbon atoms of diimines are orange-colored. View (**b**) demonstrate short π–π intermolecular contacts.

Four independent molecules form the unit cells of both compounds (Figure 2). However, their mutual arrangement displays the essential differences, leading to two types of crystal packing. Concerning complex **1**, a lamellar crystal structure is observed (Figure 3), while T-motifs can be considered as the “building blocks” of a crystal packing in the case of derivative **2** (Figure 4). In addition, there is one solvent molecule (CH_3_CN in **1**/CH_2_Cl_2_ in **2**) per one molecule of a coordination compound.

Each layer of a crystal **1** looks like a “checkerboard order”, which is represented by two types of “rows”: molecules of one located in identical positions, whereas the molecules of a neighboring layer are displaced and deployed almost perpendicularly (Figure 3, top left view). Due to such a turn, the crystal packing of **1** is built by two species of stacks, which are stabilized by intermolecular π–π contacts between π-orbitals of 2,2′-bipyridines of the neighboring molecules stacked according to the “head-to-tail” manner with a significant displacement (Figure 3, top left view). As a consequence, the distances between two diimine π-systems are equal to 3.340 Å in all stacks. It should be noticed that there are no inter-stacks contacts—the shortest distance C–C = 4.217 Å transcends possible van der Waals interactions. The lamellar crystal structure of **1** can be described as non-ideal because the planes of the nearest molecules from the same layer (i.e., the molecules of the neighboring stacks) demonstrate a tilt at 12.41° (Figure 3, top right and bottom views).

The crystal packing of **2** is folded by the perpendicular T-motifs which are formed by intermolecular π–π stacking between diimines’ π-systems (Figure 4): (1) a dihedral angle between the planes of molecules from neighboring T-motifs is 89.37° (Figure 4b); (2) the planes of neighboring molecules within one T-motif are 3.416 Å apart (planes between molecules are taken into account, excluding the C(1A) and C(2A) atoms of the glycol fragment). In contrast to derivative **1**, the inter-stacks attractive interactions stabilize the crystal structure in complex **2**. Thus, C(16)∙∙∙C_ch_ interplay is found to be equal to 3.276 Å (where C_ch_ is a centroid of the O(2)–Ni(1)–O(1)–C(1)–C(6) chelate cycle).

### 2.3. Electrochemical Study of the Complexes ***1*** and ***2***

**Figure 5:**
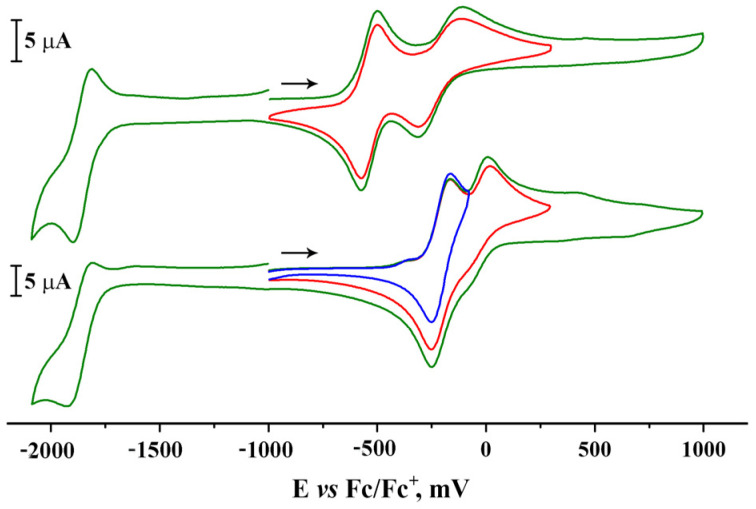
The CV data for **2** (top view) and **1** (bottom view) (GC electrode, Ag/AgCl/KCl, 0.2 M Bu_4_NClO_4_, CH_2_Cl_2_, C = 2 × 10^−3^ mol/dm^3^, V = 0.2 V·s^−1^, Ar). Corrections to Fc/Fc^+^ couple as a standard were made.

Exploring the electrochemical behavior of Ni^II^ derivatives **1** and **2** was carried out through the cyclic voltammetry method in CH_2_Cl_2_ solution applying a three-unit cell equipped with a glassy carbon working electrode. A similar ligand-centered nature characterizes observed electrochemical conversions for both compounds. In particular, the electrochemical oxidation of **1** and **2** occurs in two successive one-electron reversible stages corresponding to the transformation of a catecholate dianion to an *o*-benzosemiquinonato radical anion and a neutral *o*-benzoquinone, respectively (Figure 5). We have performed CV studies at 50, 100, 200, 300, and 400 V·s^−1^ rates. Under high scan rates, two oxidation waves for compound **2** are merge into one two-electron wave, and the potential difference decreases during the direct and reverses sweep.

**Scheme 3:**
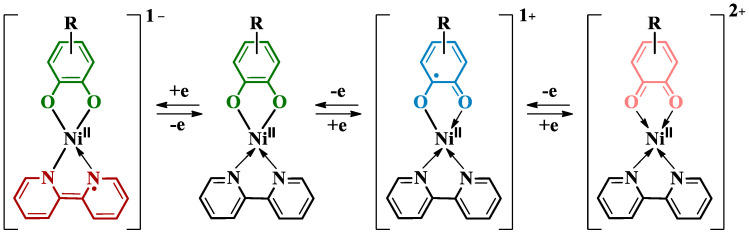
Proposed redox transformations of **1** and **2**.

On the contrary, a one-electron quasi-reversible wave exists in the cathode region of the voltammograms at −1.86 V caused by a reductive process of a 2,2′-bipyridine fragment. Proposed redox transformations are depicted in Scheme 3.

A related electrochemical behavior was described for reported Pd^II^ analog [35] of studied complex **1** (Table 2, literature data). But, both oxidative waves are shifted to the cathodic region for compounds **1** and **2** (Table 2).

Found values of the electrochemical potentials *E*_1/2_^Ox.1^ and *E*_1/2_^Ox.2^ for the complex **1** substantially exceed ones for the derivative **2** (Table 2). This fact indicates a decrease of HOMO_catecholate_ energy and, consequently, a respective lowering of the LL’CT energy in **2**, caused by higher donor features of the catechol moiety annelated with an electron donor glycol fragment.

### 2.4. UV-Vis-NIR Spectroscopy for Complexes ***1*** and ***2***

**Figure 6:**
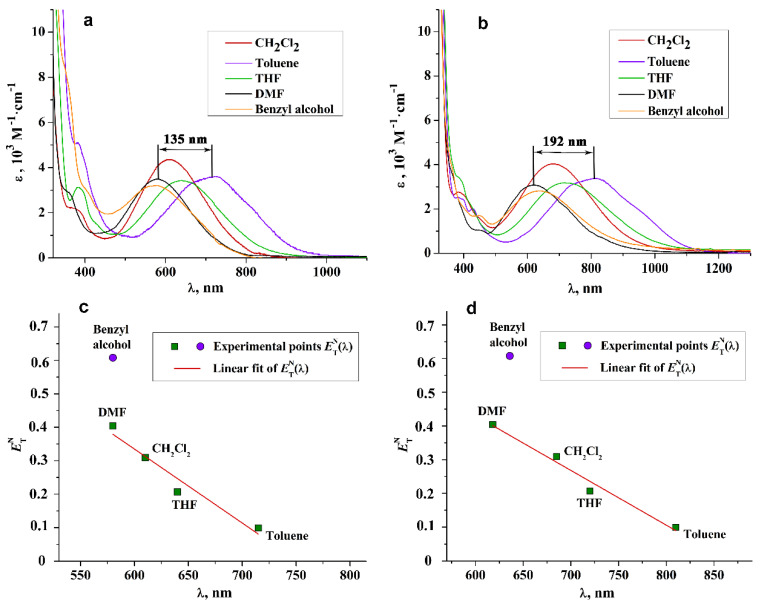
UV-Vis-NIR spectra for **1** (**a**) and **2** (**b**); *E*_T_^N^(λ) dependences accompanied with linear fits for **1** (**c**) and **2** (**d**).

The same character is inherent for UV-Vis-NIR spectra of **1** and **2**, as shown in Figure 6. Nevertheless, the presence of an electron donor glycol fragment in the catecholate ligand in **2** contributes a substantial shift of the LL’CT (HOMO_catecholate_ → LUMO_α-diimine_) absorption band to the longwave region towards **1** (the characteristics of corresponding shifts Δλ_2-1_ in the set of solvents are given in Table 3), as a consequence of HOMO_catecholate_ and LL’CT energy’s lowering. In particular, the most incredible Δλ_2-1_ shift is fixed in the toluene solution, which is equal to 95 nm. A similar perceptible value is found in THF and CH_2_Cl_2_ (80 and 75 nm, respectively). For comparison, it has been reported recently [15] the record displacements of the LL’CT absorption peaks at 206 nm in THF for the square-planar α-diimine–Ni^II^–catecholate derivatives on the base of N,N′-bis(2,4,6-trimethylphenyl)-2,3-butanediimine. However, in this case, the nature of the catecholate fragment was changed more drastically—from 3,5-di-*tert*-butyl-*o*-benzoquinone (3,5-DTBQ) to 9,10-phenanthrenequinone.

Another striking peculiarity of registered UV-Vis-NIR spectra is a pronounced negative solvatochromism towards both Ni^II^ derivatives with a considerable hypsochromic displacement Δλ_hyp_ = 135 nm (for **1**) and 192 nm (for **2**) from toluene to DMF (N,N′-dimethylformamide) (Table 3). As was mentioned in the Introduction Part, the investigations addressed to the solvatochromic features of such type coordination compounds are not numerous and are represented by sporadic works [15,17]. As a comparison, the blue LL’CT shift was observed at 175 nm (from toluene to methanol) for related Ni^II^ complex derived from 3,5-di-*tert*-butyl-*o*-benzoquinone (3,5-DTBQ) and *tert*-butyl-substituted 2,2′-bipyridine [17]. Finally, the record-breaking situation is described for a series of α-diimine–Ni^II^–catecholate species when the hypsochromic displacement reaches 400 nm approximately [15].

As shown in Figure 6c,d, the negative solvatochromic effect established for **1** and **2** correlates finely with the changes in the polarity of the solvents, which is expressed by the normalized empirical parameter *E*_T_^N^ by Dimroth and Reichardt [67]. The parameter *E*_T_^N^ characterizes the non-specific polarity of the solvent defined as a change of the electron transfer energy for a longwave band in the electronic spectrum for the standard N-phenoxypyridinium betaine dye. Thus, a decrease of the solvent polarity facilitates the lowering of energy for the non-polar excited state of the charge transfer α-diimine–Ni^II^–catecholate complexes **1** and **2**. It is noteworthy that the UV-Vis-NIR spectra recorded in benzyl alcohol do not follow the above general conception (Figure 6), which might be caused by specific solvation of **1** and **2** by this solvent against other used solvents (DMF, CH_2_Cl_2_, THF, toluene).

### 2.5. DFT Calculations

**Figure 7:**
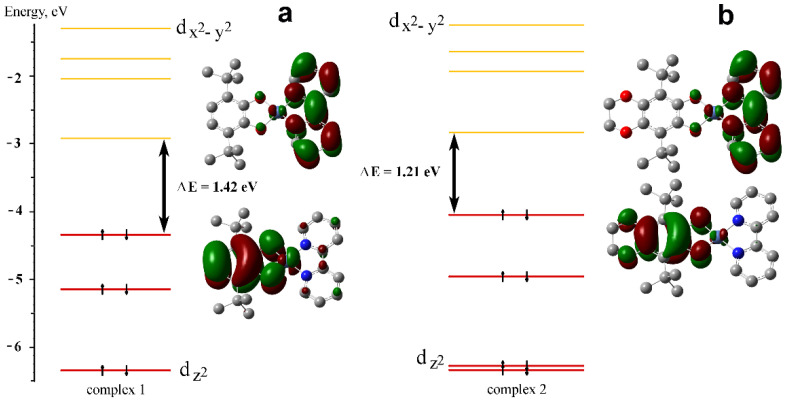
Frontier Kohn–Sham orbital diagram for complexes **1** (**a**) and **2** (**b**) according to DFT calculations at the B3LYP/Def2TZVP level of theory. Isovalue = 0.03 a.u. The H atoms are omitted for clarity.

Density functional theory (DFT) calculations were performed to investigate the electronic structure of the Ni^II^ complexes **1** and **2**. The experimental (X-ray) geometry was used as the initial one for the optimization at the B3LYP/Def2TZVP level of theory. Both compounds were calculated in closed-shell approximation with S = 0 ground state. The optimized geometries are quite close to those obtained from the structural experiments. The bond lengths in **1** and **2** differ compared to X-ray data by no more than 0.02 Å. The leading destination of these quantum-chemical calculations was to estimate the energy and view of boundary orbitals that provide redox and spectral properties of compounds under investigation. The frontier Kohn–Sham orbital diagrams with the visualization of frontier orbitals are presented in Figure 7.

Both compounds have well-isolated HOMO (−4.33 eV and 4.04 eV for **1** and **2** respectively) orbitals located entirely on the catecholate part of complexes with small nickel d_xy_ orbital and π-system of 2,2′-bipyridine fragment contribution to this MO. This HOMO determines the reductive properties of the compounds, which are mainly determined by the donor fragment. The energy of the HOMO orbital increases significantly (0.3 eV) when the glycol fragment is introduced into the 3,6-di-*tert*-butyl-catecholate ligand. The nearest filled orbital with a significant contribution of metal AO is the HOMO-2. It lies deep enough (−6.35 eV and 6.27 eV for **1** and **2,** respectively), corresponds to the d_z2_ nickel orbital, and weakly depends on the nature of the catecholate ligand. This orbital will not be affected in redox transformations. The LUMO orbitals (−2.92 eV and -2.83 eV for **1** and **2,** respectively) also have an almost purely ligand nature and occupy the acceptor diimine fragment of the complex. The LUMO energy level remains practically unchanged for complexes **1** and **2**. The LUMO+3 (−1.29 eV and −1.24 eV for **1** and **2** respectively) are predominantly nickel d_x2-y2_ with L-M σ*-bond in character and significantly higher. The catecholate ligand nature does not influence them. Thus, complexes **1** and **2** can be considered as donor-acceptor compounds in which the ligand framework provides electronic properties. The slight variation of substituents in the donor catecholate fragments leads to the significant change in HOMO energy level and respective redshift in the UV-Vis-NIR spectrum. The HOMO-LUMO gaps (1.42 and 1.21 eV for **1** and **2** respectively) calculated for compounds are in good agreement with those estimated from the electrochemical (1.65 eV and 1.32 eV for **1** and **2** respectively) data, and it reproduces well the difference observed in the electronic spectra of the studied complexes. The highest degree of HOMO orbital localization on the catecholate donor ligand for compound **2** should lead to the most remarkable difference in polarity of ground (calculated dipole moments directed from 2,2′-bipyridine to catecholate ligand are 11.59 and 9.34 Debye for **1** and **2** respectively) and excited states. It explains the much more pronounced solvatochromic effect for Ni^II^ complex **2** in comparison with **1**.

Eventually, the current work continues the evolving tendency in a molecular design of the charge transfer complexes to influence the LL’CT energy by a fine-tuning of the ligands′ composition. Thus, even the “light” enough transformation—the annellation of the catecholate part by electron donor glycol fragment—can promote an appreciable drop of HOMO–LUMO gap (≈0.2 eV) and provide an abrupt intensification of the solvatochromic nature of transition metal complex (blue shift 192 nm in **2** vs. 135 nm in **1**).

## 3. Materials and Methods

### 3.1. Reagents and Methods

The synthesis of the metal complexes **1** and **2**, registration of their IR-, ^1^H NMR-, UV-Vis-NIR spectra, and an electrochemical study was implemented under argon atmosphere in a glovebox. Prepared compounds were purified by recrystallization under reduced pressure from the same solvent systems used to grow X-ray quality single crystals.

Starting reagents 2,2′-bipyridine and Ni(cod)_2_ were commercial products and implied without additional purification. The solvents required for the experiments were purified and dehydrated according to the procedures described in the literature [67,68]. 3,6-di-*tert*-butyl-*o*-benzoquinone (**3,6-DTBQ**) [69] and 7,10-di-*tert*-butyl-2,5-dioxabicyclo(4.4.0)deca-1(10),6-diene-8,9-dione (**3,6-DTBQ^gly^**) [69] were prepared in an accordance with the previously published synthetic procedures. The elemental analysis and spectral characteristics of used *o*-benzoquinone ligands correspond to those reported earlier.

The elemental analysis was carried out applying Vario el Cube instrument. The IR-spectra of studied compounds were registered on an FSM1201 Fourier-IR spectrometer in a Nujol using KBr cuvettes in the range 4000–400 cm^−1^. The ^1^H NMR-spectrum was recorded in CDCl_3_ solution with a Bruker Avance Neo 300 MHz spectrometer. The signal positions were correlated with TMS as a standard. The UV-Vis-NIR spectra were obtained on Shimadzu UV-3600 spectrophotometer with quartz cuvettes with path length l = 1 cm at the interval 250–1700 nm. The cyclic voltammetry (CV) measurements were performed for 2∙10^−3^ M solutions of **1** and **2** (CH_2_Cl_2_, 0.2 M Bu_4_NClO_4_) with the 0.2 V∙s^−1^ rate scan by applying the three-electrode cell: Ag/AgCl/KCl reference electrode, platinum-flag auxiliary electrode, and a glassy carbon working electrode. The potential of the Fc/Fc^+^ redox pair was assigned as an internal standard to evaluate the number of electrons that were transported during the electrode procedure.

### 3.2. Single-Crystal X-ray Diffraction Studies

The X-ray diffraction for the complex **1** was measured on a Bruker D8 Venture Photon single crystal diffractometer equipped with microfocus sealed tube Incoatec IµS 3.0 (Mo *Kα* radiation, λ = 0.71073 Å) in *φ*- and *ω*-scan mode at the Center of shared equipment IGIC RAS. The raw data for **1** were treated with the *APEX3* program suite [70]; experimental intensities were corrected for absorption effects using the *SADABS* program [70]. X-ray diffraction data for the complex **2** were collected on the “Belok” beamline at the Kurchatov Synchrotron Radiation Source (National Research Center “Kurchatov Institute”, Moscow, Russian Federation) in *φ*-scan mode using Rayonix SX165 CCD detector, λ = 0.74539 Å [71]. The raw data for **2** were treated with the *XDS* data reduction program [72], including absorption correction.

The crystal structures were solved by direct methods [73] and refined by the full-matrix least-squares on *F^2^* [74]. All non-hydrogen atoms were refined using anisotropic displacement parameters without any restraints. The hydrogen atoms were placed in the ideal calculated positions and refined using the riding model with *U*_iso_(H) = 1.5*U*_eq_(C) for the methyl groups and with *U*_iso_(H) = 1.2*U*_eq_(C) for other hydrogen atoms.

Both low-temperature X-ray diffraction experiments were carried out using an Oxford Cryosystems Cryostream 800 open-system cooler. Crystal data and structure refinement details for **1** and **2** are presented in Table 4.

### 3.3. The Synthetic Procedure for the Complexes ***1*** and ***2***

Complex [Ni^II^(3,6-Cat)(bipy)]∙CH_3_CN (**1**)**.** A solution of 2,2′-bipyridine (0.28 g, 1.8 mmol) in toluene (10 mL) was poured to an equimolar amount of Ni(cod)_2_ (0.5 g, 1.8 mmol) in the same solvent (20 mL). The resulting reaction mixture was stirred in the ampoule for 5 h. In the course of this time, the solution became a dark purple. Next, an equimolar amount of **3,6-DTBQ** (0.4 g, 1.8 mmol) in toluene (10 mL) was added to the resulting solution. The color of the reaction mixture turned to dirty-green with the formation of a small amount of a dark precipitate. It was filtered under reduced pressure using a glass filter. After that, the solvent was evaporated and changed to CH_2_Cl_2_ (15 mL), producing a dark blue resulting solution. X-ray quality dark blue needle-shaped single crystals of compound **1** were grown by a slow evaporating of the solvent mixture CH_2_Cl_2_/CH_3_CN (3:1, vol.) under reduced pressure and further storage for one day. The total yield is 0.54 g (68%) based on the starting ligand.

Anal. Calc. for the solvent-free complex **1** with C_24_H_28_N_2_NiO_2_ composition (%): C, 66.24; H, 6.49; N, 6.44. Found: C, 65.89; H, 6.17; N, 6.24.

^1^H NMR (300 MHz, CDCl_3_, 20 °C, δ/ppm): 1.23 (*^t^*Bu, 9H), 1.47 (*^t^*Bu, 9H), 5.29 (CH_Ar_, 4H), 6.31 (CH_Ar_, 1H), 6.75 (CH_Ar_, 1H), 7.46 (CH_Ar_, 1H), 7.79 (CH_Ar_, 1H), 7.95 (CH_Ar_, 1H), 8.86 (CH_Ar_, 1H).

IR (Nujol, KBr) cm^−1^: 465 (m), 528 (w), 615 (m), 650 (m), 673 (w), 719 (s), 742 (w), 764 (s), 780 (m), 799 (w), 812 (m), 892 (m), 904 (w), 926 (m), 943 (m), 983 (s), 1025 (m), 1031 (w), 1045 (w), 1053 (w), 1085 (m), 1119 (w), 1142 (w), 1155 (m), 1204 (m), 1247 (s), 1260 (w), 1273 (s),1307 (m), 1319 (w), 1351 (w), 1410 (s), 1421 (s), 1450 (m), 1566 (w), 1594 (w),1606 (m), 1652 (w), 1680 (w), 1707 (w), 1741 (m), 1771 (w), 1803 (w), 1857 (w), 1867 (w), 1894 (w), 1961 (w), 1982 (w), 2006 (w), 3055 (w), 3078 (w), 3090 (m).

Complex [Ni^II^(3,6-Cat^gly^)(bipy)]∙CH_2_Cl_2_ (**2**)**.** The same synthetic procedure was applied for obtaining complex **2.** The following quantities of initial reactants were used: 2,2′-bipyridine (0.28 g, 1.8 mmol), Ni(cod)_2_ (0.5 g, 1.8 mmol), **3,6-DTBQ^gly^** (0.51 g, 1.8 mmol). After the end of the reaction, there was a dirty-green toluene solution. Further filtration and exchange of solvent to CH_2_Cl_2_ turned the color of the resulting mixture to an emerald tone. Plate-shaped green single crystals of compound **2**, suitable for X-ray diffraction analysis, were grown from a CH_2_Cl_2_/hexane (3:1, vol.) mixture by a slow evaporating of the solvents under reduced pressure. The total yield is 0.68 g (76%) based on the starting ligand.

Anal. Calc. for the solvent-free complex **2** with C_26_H_30_N_2_NiO_4_ composition (%): C, 63.31; H, 6.13; N, 5.68. Found: C, 62.91; H, 5.87; N, 5.75.

^1^H NMR-spectrum of complex **2** in CDCl_3_ is broadened substantially at T = 20 °C, and signals observed cannot be assigned essentially. Double recrystallization of the resulting compound does not change the situation. Probably it is caused by the oxidation of complex **2** by CDCl_3_ to produce paramagnetic derivatives. The solubility of **2** in benzene is not sufficient to record NMR spectra. Another probable reason for such a strong broadening of resonances is the tetrahedral distortion of complex in solution. This issue can be an object of further investigation.

IR (Nujol, KBr) cm^−1^: 459 (w), 499 (w), 516 (w), 555 (w), 586 (m), 636 (w), 660 (m), 680 (w), 721 (m), 737 (w), 753 (s), 782 (w), 800 (w), 809 (w), 880 (m), 921 (w), 943 (m), 969 (m), 992 (m), 1042 (w), 1051 (w), 1112 (s), 1132 (w), 1157 (w), 1197 (m), 1225 (w), 1237 (m), 1265 (m), 1308 (w), 1336 (m), 1398 (s), 1549 (w), 1568 (w), 1579 (w), 1607 (m), 1741 (w), 3079 (w), 3088 (w).

### 3.4. DFT Calculations

Density functional theory (DFT) calculations were performed using the Gaussian 09 program package [75] at the B3LYP/ Def2TZVP level. The applied approximation was recently shown [76,77] to accurately reproduce the geometry, electronic, and energy characteristics of metal complexes with redox-active ligands. The stationary points on the potential energy surfaces were located by full geometry optimization with the calculation of the force constant matrix and checking for the stabilities of the DFT wave function. Structural visualizations in Figure 7 were produced with the ChemCraft program suite [78].

## 4. Conclusions

Two square-planar LL’CT chromophores [Ni^II^(3,6-Cat)(bipy)]∙CH_3_CN (**1**) and [Ni^II^(3,6-Cat^gly^)(bipy)]∙CH_2_Cl_2_ (**2**) of the α-diimine-Ni^II^-catecholate type were synthesized and studied comprehensively in terms of the molecular/electronic structure, and the electrochemical/spectral behavior.

Present research demonstrated unambiguously that the fine-tuning molecular design towards the donor part of LL’CT dyes can significantly affect the HOMO_catecholate_ → LUMO_α-diimine_ charge transfer energy and solvatochromic properties. In particular, the “light” modification, such as the annelation of catecholate ligand with an electron donor glycol fragment, led to (1) an appreciable narrowing of the HOMO–LUMO gap; (2) a considerable hypsochromic shift of the longwave peaks in the UV-Vis-NIR spectra; (3) a greater sensitivity of excited state of complex towards a solvent polarity due to a more effective LL’CT with lower energy.

Thus, the achieved results confirm the advisability of further investigations aimed at a targeted design of donor parts of α-diimine-Ni^II^-catecholate derivatives. In prospect, it will be possible to create effective materials for photovoltaics or optics applications due to a synergy of various directions in the molecular design of LL’CT dyes. Modifying the acceptor diimine part by electron-withdrawing anchor functions allows introducing compound to the semiconductor layer, substrate application, etc. A tuning of donor part by electron donor substituents for an effective charge separation improves the spectral parameters (the position and width of the bandgap) of α-diimine-Ni^II^-catecholate LL’CT chromophores.

## Figures and Tables

**Table 1:** Selected bond lengths (Å) and angles (°) for **1** and **2**.

Bond	1	2
Ni(1)–O(1)	1.814(3)	1.818(2)
Ni(1)–O(2)	1.815(3)	1.814(3)
Ni(1)–N(1)	1.882(4)	1.883(3)
Ni(1)–N(2)	1.891(3)	1.887(3)
O(1)–C(1)	1.348(5)	1.368(4)
O(2)–C(6)	1.365(5)	1.360(4)
C(1)–C(2)	1.422(6)	1.410(4)
C(2)–C(3)	1.398(6)	1.408(5)
C(3)–C(4)	1.379(6)	1.410(5)
C(4)–C(5)	1.405(6)	1.410(5)
C(5)–C(6)	1.406(6)	1.400(5)
C(1)–C(6)	1.397(6)	1.406(5)
N(1)–C(15)	1.352(5)	1.335(5)
N(1)–C(19)	1.360(5)	1.357(4)
N(2)–C(20)	1.365(5)	1.363(4)
N(2)–C(24)	1.333(6)	1.337(5)
**Angle**	**1**	**2**
O(1)–Ni(1)–O(2)	88.78(14)	88.43(11)
O(1)–Ni(1)–N(1)	177.57(15)	178.39(13)
O(2)–Ni(1)–N(1)	93.46(15)	93.17(13)
O(1)–Ni(1)–N(2)	93.73(16)	94.75(12)
O(2)–Ni(1)–N(2)	177.49(17)	176.78(13)
N(1)–Ni(1)–N(2)	84.03(17)	83.65(13)

**Table 2:** The CV data for **1** and **2** and a comparison with the literature data (potentials are given vs. Fc/Fc^+^ redox couple).

Compound	*E*_1/2_^Red.1^, V	*E*_1/2_^Ox.1^, V	*E*_1/2_^Ox.2^, V
**1**	−1.86	−0.21	−0.03
**2**	−1.86	−0.54	−0.21
**[Pd]** [35]	−1.80	−0.18	0.70

**Table 3:** The characteristics of the LL’CT peaks for **1** and **2**.

	Solvent	1		2		Shift (2-1)	
Shift (hyp)		λ_max_, nm (ε, 10^3^ M^−1^·cm^−1^)	E, eV	λ_max_, nm (ε, 10^3^ M^−1^·cm^−1^)	E, eV	Δλ_2-1_, nm	ΔE_2-1_, eV
DMF		580 (3.50)	2.138	618 (3.08)	2.007	38	0.131
CH_2_Cl_2_		610 (4.35)	2.033	685 (4.04)	1.810	75	0.223
THF		640 (3.43)	1.938	720 (3.18)	1.722	80	0.216
Toluene		715 (3.57)	1.734	810 (3.38)	1.531	95	0.203
Benzyl alcohol		580 (3.21)	2.138	636 (2.82)	1.950	56	0.188
Δλ_hyp_, nm		135		192			
ΔE_hyp_, eV		0.404		0.476			

**Table 4:** X-ray crystallographic data and structure refinement for **1** and **2**.

Parameter	1∙CH_3_CN	2∙CH_2_Cl_2_
Formula	C_26_H_31_N_3_NiO_2_	C_27_H_32_Cl_2_N_2_NiO_4_
Formula weight	476.25	578.15
Temperature (K)	100(2)	100(2)
Radiation source	microsource	synchrotron
wavelength, Å	0.71073	0.74539
colour, habit	blue, needle	green, plate
Crystal size, mm	0.140 × 0.030 × 0.015	0.200 × 0.080 × 0.015
Crystal system	Monoclinic	Monoclinic
Space group	*P*2_1_/c	*P*2_1_/c
Unit cell dimentions		
*a*, Å	6.8087(13)	16.283(7)
*b*, Å	13.788(3)	9.7500(5)
*c*, Å	25.089(4)	17.165(3)
β, deg	90.403(5)	99.12(3)
*V*, Å^3^	2355.3(8)	2690.6(13)
*Z*	4	4
density (calcd), g/cm^3^	1.343	1.427
Absorption coefficient (mm^−1^)	0.851	1.078
F(000)	1008	1208
*θ* range, deg	2.195–25.058	1.328–31.018
Index ranges	−8 ≤ h ≤ 8, −15 ≤ k ≤ 16, −29 ≤ l ≤ 29	−22 ≤ h ≤ 22, −13 ≤ k ≤ 13, −23 ≤ l ≤ 23
Reflections collected	15,342	31,754
Independent reflections	4087 [*R*_int_ = 0.1175]	7442 [*R*_int_ = 0.1080]
Data/restraints/parameters	4087/0/296	7442/0/332
*R*_1_, w*R*_2_ [I > 2σ(I)]	0.0599, 0.0963	0.0607, 0.1409
*R*_1_, w*R*_2_ (all data)	0.1428, 0.1176	0.1163, 0.1675
goodness-of-fit on *F*^2^	0.989	1.034
*T*_min_/*T*_max_	0.5897/0.7454	0.001/1.000
Extinction coefficient	−	0.0024(5)
Δρ_max_/Δρ_min_ (e/Å^3^)	0.523/−0.492	0.679/−1.348

## Data Availability

The data presented in this study are available in the article and supplementary materials. Copies of the above information may be received free of charge from The Director, CCDC, 12, Union Road, Cambridge CB2 1EZ, U.K.; fax +44-1223-336033; e-mail deposit@ccdc.cam.ac.uk.

## Supplementary Materials

The following are available online https://www.ccdc.cam.ac.uk/solutions/data/, CIF and CheckCIF files for **1** and **2**. Crystallographic data for the structural analysis has been deposited with the Cambridge Crystallographic Data Centre, CCDC No. 2086761 (**1**), 2086762 (**2**).

## Sample Availability

Samples of compounds **1** and **2** are available from the authors.

## References

[1] Ward M.D. (2005). Near-infrared electrochromic materials for optical attenuation based on transition-metal coordination complexes. J. Solid State Electrochem..

[2] Atallah H., Taliaferro C.M., Wells K.A., Castellano F.N. (2020). Photophysics and ultrafast processes in rhenium(I) diimine dicarbonyls. Dalton Trans..

[3] García-Cañadas J., Meacham A.P., Peter L.M., Ward M.D. (2003). A Near-Infrared Electrochromic Window Based on an Sb-Doped SnO_2_ Electrode Modified with a Ru–Dioxolene Complex. Angew. Chem. Int. Ed..

[4] Mukherjee S., Weyhermüller T., Bill E., Wieghardt K., Chaudhuri P. (2005). Tuning of spin transition in radical-containing iron (III) complexes by remote ligand substituents. Inorg. Chem..

[5] Chun H., Verani C.N., Chaudhuri P., Bothe E., Bill E., Weyhermüller T., Wieghardt K. (2001). Molecular and electronic structure of octahedral o-aminophenolato and o-iminobenzosemiquinonato complexes of V (V), Cr (III), Fe (III), and Co (III). Experimental determination of oxidation levels of ligands and metal ions. Inorg. Chem..

[6] Ye S., Sarkar B., Lissner F., Schleid T., van Slageren J., Fiedler J., Kaim W. (2005). Three-Spin System with a Twist: A Bis (semiquinonato) copper Complex with a Nonplanar Configuration at the Copper (II) Center. Angew. Chem. Int. Ed..

[7] Bubrin M., Paretzki A., Hübner R., Beyer K., Schwederski B., Neugebauer P., Zalis S., Kaim W. (2017). Probing the Intramolecular Metal-Selenoether Interaction in a Bis (iminosemiquinone) copper (II) Compound. Z. Anorg. Allg. Chem..

[8] Rakshit R., Ghorai S., Biswas S., Mukherjee C. (2014). Effect of ligand substituent coordination on the geometry and the electronic structure of Cu (II)-diradical complexes. Inorg. Chem..

[9] Benedix R., Hennig H., Kunkely H., Vogler A. (1990). Optical ligand-to-ligand charge transfer of Zn(2,2′-bipyridyl)(3,4-toluenedithiolate). Chem. Phys. Lett..

[10] Cameron L.A., Ziller J.W., Heyduk A.F. (2016). Near-IR absorbing donor–acceptor ligand-to-ligand charge-transfer complexes of nickel (II). Chem. Sci..

[11] Brasse M., Campora J., Davies M., Teuma E., Palma P., Alvarez E., Sanz E., Reyes M.L. (2007). Integrating Catalyst and Co-Catalyst Design in Olefin Polymerization Catalysis: Transferable Dianionic Ligands for the Activation of Late Transition Metal Polymerization Catalysts. Adv. Synth. Catal..

[12] Tahara K., Ashihara Y., Higashino T., Ozawa Y., Kadoya T., Sugimoto K., Ueda A., Mori H., Abe M. (2019). New π-extended catecholato complexes of Pt(II) and Pd(II) containing a benzothienobenzothiophene (BTBT) moiety: Synthesis, electrochemical behavior and charge transfer properties. Dalton Trans..

[13] BaniKhaled M.O., Becker J.D., Koppang M., Sun H. (2016). Perfluoroalkylation of Square-Planar Transition Metal Complexes: A Strategy To Assemble Them into Solid State Materials with a π–π Stacked Lamellar Structure. Cryst. Growth Des..

[14] Yang H., Zhao Y., Liu B., Su J.-H., Fedushkin I.L., Wu B., Yang X.-J. (2017). Noninnocent ligands: Heteroleptic nickel complexes with α-diimine and 1,2-diketone derivatives. Dalton Trans..

[15] Kramer W.W., Cameron L.A., Zarkesh R.A., Ziller J.W., Heyduk A.F. (2014). Donor–Acceptor Ligand-to-Ligand Charge-Transfer Coordination Complexes of Nickel (II). Inorg. Chem..

[16] Bubnov M.P., Teplova I.A., Druzhkov N.O., Fukin G.K., Cherkasova A.V., Cherkasov V.K. (2015). Catecholato complexes of cobalt and nickel with 1,4-disubstituted-1,4-diazabutadiens-1,3 and 1,2-bis(diphenylphosphino)ethane. J. Chem. Sci..

[17] Yamada S., Matsumoto T., Chang H.C. (2019). Impact of Group 10 Metals on the Solvent-Induced Disproportionation of o-Semiquinonato Complexes. Chem. Eur. J..

[18] Kokatam S.L., Chaudhuri P., Weyhermüller T., Wieghardt K. (2007). Molecular and electronic structure of square planar complexes [Pd^II^(^t^bpy)(L^IP^_N,O_)]^0^, [Pd^II^(^t^bpy)(L^ISQ^_N,O_)](PF_6_), and [Pd^II^(^t^bpy)(L^IBQ^_N,O_)](PF_6_)(BF_4_)·2CH_2_Cl_2_: An o-iminophenolato based ligand centered, three-membered redox series. Dalton Trans..

[19] Best J., Sazanovich I.V., Adams H., Bennett R.D., Davies E.S., Meijer A.J.H.M., Towrie M., Tikhomirov S.A., Bouganov O.V., Ward M.D. (2010). Structure and ultrafast dynamics of the charge-transfer excited state and redox activity of the ground state of mono-and binuclear platinum (II) diimine catecholate and bis-catecholate complexes: A transient absorption, TRIR, DFT, and electrochemical study. Inorg. Chem..

[20] Heinze K., Reinhardt S. (2008). Platinum (II) Complexes with Non-Innocent Ligands: Solid-Phase Synthesis, Redox Chemistry and Luminescence. Chem. Eur. J..

[21] Sobottka S., Nößler M., Ostericher A.L., Hermann G., Subat N.Z., Beerhues J., Behr-van der Meer M., Suntrup L., Albold U., Hohloch S. (2020). Tuning Pt^II^-Based Donor–Acceptor Systems through Ligand Design: Effects on Frontier Orbitals, Redox Potentials, UV/Vis/NIR Absorptions, Electrochromism, and Photocatalysis. Chem. Eur. J..

[22] Scattergood P.A., Jesus P., Adams H., Delor M., Sazanovich I.V., Burrows H.D., Serpa C., Weinstein J.A. (2015). Exploring excited states of Pt(II) diimine catecholates for photoinduced charge separation. Dalton Trans..

[23] Shavaleev N.M., Davies E.S., Adams H., Best J., Weinstein J.A. (2008). Platinum (II) diimine complexes with catecholate ligands bearing imide electron-acceptor groups: Synthesis, crystal structures,(spectro) electrochemical and EPR studies, and electronic structure. Inorg. Chem..

[24] Yang J., Kersi D.K., Giles L.J., Stein B.W., Feng C., Tichnell C.R., Shultz D.A., Kirk M.L. (2014). Ligand control of donor–acceptor excited-state lifetimes. Inorg. Chem..

[25] Okabe N., Hagihara K., Odoko M., Muranishi Y. (2004). (2,2′-Bipyridine-κ^2^N,N′)(2,3-naphthalenediolato-κ^2^O,O′) palladium(II) and (2,2′-biquinoline-κ^2^N,N′)(2,3-naphthalenediolato-κ^2^O,O′) palladium(II). Acta Crystallogr..

[26] Okabe N., Muranishi Y., Aziyama T. (2003). (1,2-Benzenediolato-κ^2^O,O′)(1,10-phenanthroline-κ^2^N,N′) palladium(II) dihydrate. Acta Crystallogr..

[27] Okabe N., Aziyama T., Odoko M. (2005). (1,2-Benzenediolato-κ^2^O,O′)(2,2′-biquinoline-κ^2^N, N′) palladium (II). Acta Crystallogr..

[28] Pierpont C.G. (2011). Ligand redox activity and mixed valency in first-row transition-metal complexes containing tetrachlorocatecholate and radical tetrachlorosemiquinonate ligands. Inorg. Chem..

[29] Brown S.N. (2012). Metrical Oxidation States of 2-Amidophenoxide and Catecholate Ligands: Structural Signatures of Metal–Ligand π Bonding in Potentially Noninnocent Ligands. Inorg. Chem..

[30] Poddel’sky A.I., Cherkasov V.K., Abakumov G.A. (2009). Transition metal complexes with bulky 4,6-di-tert-butyl-N-aryl(alkyl)-o-iminobenzoquinonato ligands: Structure, EPR and magnetism. Coord. Chem. Rev..

[31] Kaim W., Paretzki A. (2017). Interacting metal and ligand based open shell systems: Challenges for experiment and theory. Coord. Chem. Rev..

[32] Mao G., Song Y., Hao T., Li Y., Xu T., Zhang H., Jiang T. (2014). Progress in the research of radical anion ligands and their complexes. Chin. Sci. Bull..

[33] Kaim W. (2011). Manifestations of noninnocent ligand behavior. Inorg. Chem..

[34] Butin K.P., Beloglazkina E.K., Zyk N.V. (2005). Metal complexes with non-innocent ligands. Russ. Chem. Rev..

[35] Ghosh P., Begum A., Herebian D., Bothe E., Hildenbrand K., Weyhermüller T., Wieghardt K. (2003). Coordinated *o*-Dithio-and *o*-Iminothiobenzosemiquinonate(^1−^) π-Radicals in [M^II^(bpy)(L.)](PF_6_) Complexes. Angew. Chem. Int. Ed..

[36] Deibel N., Schweinfurth D., Fiedler J., Záliš S., Sarkar B. (2011). Isomeric separation in donor–acceptor systems of Pd(II) and Pt(II) and a combined structural, electrochemical and spectroelectrochemical study. Dalton Trans..

[37] Rauth G.K., Pal S., Das D., Sinha C., Slawin A.M., Woollins J.D. (2001). Synthesis, spectral characterization and electrochemical studies of mixed-ligand complexes of platinum (II) with 2-(arylazo) pyridines and catechols. Single-crystal X-ray structure of dichloro {2-(phenylazo) pyridine} platinum (II). Polyhedron.

[38] Roy R., Chattopadhyay P., Sinha C., Chattopadhyay S. (1996). Synthesis, spectral and electrochemical studies of arylazopyridine complexes of palladium (II) with dioxolenes. Polyhedron.

[39] Liu W., Heinze K. (2010). Rhenium (I) and platinum (II) complexes with diimine ligands bearing acidic phenol substituents: Hydrogen-bonding, acid–base chemistry and optical properties. Dalton Trans..

[40] Mansuri-Torshizi H., Ghadimy S., Akbarzadeh N. (2001). Synthesis, characterization, DNA binding and cytotoxic studies of platinum (II) and palladium (II) complexes of the 2, 2’-bipyridine and an anion of 1, 1-cyclobutanedicarboxylic acid. Chem. Pharm. Bull..

[41] Afrasiabi Z., Sinn E., Chen J., Ma Y., Rheingold A.L., Zakharov L.N., Rath N., Padhye S. (2004). Appended 1,2-naphthoquinones as anticancer agents 1: Synthesis, structural, spectral and antitumor activities of ortho-naphthaquinone thiosemicarbazone and its transition metal complexes. Inorg. Chim. Acta.

[42] Rebolledo A.P., Vieites M., Gambino D., Piro O.E., Castellano E.E., Zani C.L., Souza-Fagundes E.M., Teixiera L.R., Batista A.A., Beraldo H. (2005). Palladium (II) complexes of 2-benzoylpyridine-derived thiosemicarbazones: Spectral characterization, structural studies and cytotoxic activity. J. Inorg. Biochem..

[43] Padhye S., Afrasiabi Z., Sinn E., Fok J., Mehta K., Rath N. (2005). Antitumor metallothiosemicarbazonates: Structure and antitumor activity of palladium complex of phenanthrenequinone thiosemicarbazone. Inorg. Chem..

[44] Okabe N., Muranishi Y., Aziyama T. (2006). (Benzene-1,2-diolato-κ^2^O,O′)(di-2-pyridylamine-κ^2^N,N′) palladium(II) hemihydrate. Acta Crystallogr..

[45] Wang Y., Mizubayashi Y., Odoko M., Okabe N. (2005). (Di-2-pyridylamine-κ^2^N,N′)(naphthalene-2,3-diolato-κ^2^O,O′) palladium(II) monohydrate and (di-2-pyridylamine-κ^2^N,N′)(3-oxidonaphthalene-2-carboxylato-κ^2^O,O′)palladium(II). Acta Crystallogr..

[46] Okabe N., Aziyama T., Odoko M. (2005). (Benzene-1,2-diolato-κ^2^O,O′)(2,2′-bipyridine-κ^2^N,N′) palladium(II). Acta Crystallogr..

[47] Giribabu L., Kanaparthi R.K., Velkannan V. (2012). Molecular engineering of sensitizers for dye-sensitized solar cell applications. Chem. Rec..

[48] Sekar N., Gehlot V.Y. (2010). Metal complex dyes for dye-sensitized solar cells: Recent developments. Resonance.

[49] Michaels H., Benesperi I., Edvinsson T., Muñoz-Garcia A.B., Pavone M., Boschloo G., Freitag M. (2018). Copper complexes with tetradentate ligands for enhanced charge transport in dye-sensitized solar cells. Inorganics.

[50] Smirnova E.A., Besedina M.A., Karushev M.P., Vasil’ev V.V., Timonov A.M. (2016). Photogalvanic and photovoltaic effects in systems based on metal complexes of Schiff bases. Russ. J. Phys. Chem. A.

[51] O’regan B., Grätzel M. (1991). A low-cost, high-efficiency solar cell based on dye-sensitized colloidal TiO_2_ films. Nature.

[52] Grätzel M. (2005). Solar energy conversion by dye-sensitized photovoltaic cells. Inorg. Chem..

[53] Imer A.G., Syan R.H.B., Gülcan M., Ocak Y.S., Tombak A. (2018). The novel pyridine based symmetrical Schiff base ligand and its transition metal complexes: Synthesis, spectral definitions and application in dye sensitized solar cells (DSSCs). J. Mater. Sci. Mater. Electron..

[54] Neuthe K., Popeney C.S., Bialecka K., Hinsch A., Sokolowski A., Veurmann W., Haag R. (2014). Simple NIR complexes and their applicability in dye-sensitized solar cells. Polyhedron.

[55] Gauthier S., Caro B., Robin-Le Guen F., Bhuvanesh N., Gladysz J.A., Wojcik L., Le Poul N., Planchat A., Pellegrin Y., Blart E. (2014). Synthesis, photovoltaic performances and TD-DFT modeling of push–pull diacetylide platinum complexes in TiO_2_ based dye-sensitized solar cells. Dalton Trans..

[56] Liu Q., Zhu N., Ho C.L., Fu Y., Lau W.S., Xie Z., Wang L., Wong W.Y. (2016). Synthesis, characterization, photophysical and photovoltaic properties of new donor–acceptor platinum (II) acetylide complexes. J. Organomet. Chem..

[57] Islam A., Sugihara H., Hara K., Singh L.P., Katoh R., Yanagida M., Takahashi Y., Murata S., Arakawa H. (2000). New platinum(II) polypyridyl photosensitizers for TiO_2_ solar cells. New J. Chem..

[58] Kee J.W., Ng Y.Y., Kulkarni S.A., Muduli S.K., Xu K., Ganguly R., Lu Y., Hirao H., Soo H.S. (2016). Development of bis (arylimino) acenaphthene (BIAN) copper complexes as visible light harvesters for potential photovoltaic applications. Inorg. Chem. Front..

[59] Miao Q., Gao J., Wang Z., Yu H., Luo Y., Ma T. (2011). Syntheses and characterization of several nickel bis (dithiolene) complexes with strong and broad Near-IR absorption. Inorg. Chim. Acta.

[60] Linfoot C.L., Richardson P., McCall K.L., Durrant J.R., Morandeira A., Robertson N. (2011). A nickel-complex sensitiser for dye-sensitised solar cells. Sol. Energy.

[61] Son H.J., Wang W., Xu T., Liang Y., Wu Y., Li G., Yu L. (2011). Synthesis of fluorinated polythienothiophene-co-benzodithiophenes and effect of fluorination on the photovoltaic properties. J. Am. Chem. Soc..

[62] Bubnov M.P., Teplova I.A., Cherkasova A.V., Baranov E.V., Fukin G.K., Romanenko G.V., Bogomyakov A.S., Starikov A.G., Cherkasov V.K., Abakumov G.A. (2019). Metal-ligand ferromagnetic exchange interactions in heteroligand bis-o-semiquinonato nickel complexes with 2, 2′-dipyridine and 1, 10-phenanthroline. Polyhedron.

[63] Sarkar B., Hübner R., Pattacini R., Hartenbach I. (2009). Combining two non-innocent ligands in isomeric complexes [Pt(pap)_m_Q_n_]^0^ (pap = phenylazopyridine, Q = 3,5-di-tert-butyl-benzoquinone). Dalton Trans..

[64] Deibel N., Schweinfurth D., Hohloch S., Fiedler J., Sarkar B. (2012). Donor–acceptor systems of Pt(II) and redox-induced reactivity towards small molecules. Chem. Commun..

[65] Okuniewski A., Rosiak D., Chojnacki J., Becker B. (2015). Coordination polymers and molecular structures among complexes of mercury(II) halides with selected 1-benzoylthioureas. Polyhedron.

[66] Yang L., Powell D.R., Houser R.P. (2007). Structural variation in copper(I) complexes with pyridylmethylamide ligands: Structural analysis with a new four-coordinate geometry index, τ4. Dalton Trans..

[67] Reichardt C., Welton T., Reichardt C., Welton T. (2011). Solvents and Solvent Effects in Organic Chemistry.

[68] Gordon A., Ford R. (1972). The Chemist’s Companion.

[69] Fukin G.K., Cherkasov A.V., Shurygina M.P., Druzhkov N.O., Kuropatov V.A., Chesnokov S.A., Abakumov G.A. (2010). Geometrical and energetical aspects of structure of 3,6-di-tert-butyl-o-benzoquinones. Struct. Chem..

[70] Bruker (2016). APEX3, SAINT and SADABS.

[71] Svetogorov R.D., Dorovatovskii P.V., Lazarenko V.A. (2020). Belok/XSA diffraction beamline for studying crystalline samples at Kurchatov Synchrotron Radiation Source. Cryst. Res. Technol..

[72] Kabsch W. (2010). XDS. Acta Crystallogr..

[73] Sheldrick G.M. (2015). SHELXT–Integrated space-group and crystal-structure determination. Acta Crystallorg..

[74] Sheldrick G.M. (2015). Crystal structure refinement with SHELXL. Acta Crystallogr..

[75] Frisch M.J., Trucks G.W., Schlegel H.B., Scuseria G.E., Robb M.A., Cheeseman J., Scalmani R., Barone V., Mennucci B., Petersson G.A. (2013). GAUSSIAN 09. Revision D.01.

[76] Shangin P.G., Krylova I.V., Lalov A.V., Kozmenkova A.Y., Saverina E.A., Buikin P.A., Korlyukov A.A., Starikova A.A., Nikolaevskaya E.N., Egorov M.P. (2021). Supramolecular D/A-layered structures based on germanium complexes with 2,3-dihydroxynaphthalene and N,N-bidentate ligands. RSC Adv..

[77] Arsenyeva K.V., Pashanova K.I., Trofimova O.Y., Ershova I.V., Chegerev M.G., Starikova A.A., Cherkasov A.V., Syroeshkin M.A., Kozmenkova A.Y., Piskunov A.V. (2021). O,N-Heterocyclic germylenes as efficient catalysts for hydroboration and cyanosilylation of benzaldehyde. New J. Chem..

[78] (2014). Chemcraft, Version 1.8. http://www.chemcraftprog.com.

